# Psychological and Physical Intimate Partner Aggression Are Associated with Broad and Specific Internalizing Symptoms during Pregnancy

**DOI:** 10.3390/ijerph19031662

**Published:** 2022-01-31

**Authors:** Gabriela R. Perez, Sara M. Stasik-O’Brien, Lauren M. Laifer, Rebecca L. Brock

**Affiliations:** 1 Department of Psychology, University of Nebraska-Lincoln, Lincoln, NE 68588, USA; gabrielaperez@isu.edu (G.R.P.); llaifer@huskers.unl.edu (L.M.L.); 2 Department of Psychology, Idaho State University, Pocatello, ID 83209, USA; 3 Department of Psychology, Knox College, Galesburg, IL 61401, USA; smobrien@knox.edu

**Keywords:** pregnancy, perinatal mental health, couples, intimate partner aggression, psychological aggression, internalizing problems

## Abstract

Background: Intimate partner violence (IPV) has serious consequences, particularly during high-risk periods such as pregnancy, which poses a significant risk to maternal mental health. However, it is unclear whether IPV presents a broad risk for psychopathology or is specific to distinct diagnoses or symptom dimensions (e.g., panic, social anxiety). Further, the relative impact of physical versus psychological aggression remains unclear. Methods: One hundred and fifty-nine pregnant couples completed surveys assessing psychological and physical intimate partner aggression unfolding in the couple relationship, as well as a range of internalizing symptoms. Results: Psychological and physical aggression were each associated with broad negative affectivity, which underlies mood and anxiety disorders; however, only psychological aggression demonstrated a unique association. Further, for pregnant women, aggression was uniquely associated with several symptom dimensions characteristic of PTSD. In contrast, men demonstrated a relatively heterogeneous symptom presentation in relation to aggression. Conclusion: The present study identifies unique symptom manifestations associated with IPV for couples navigating pregnancy and suggests psychological aggression can be more detrimental to mental health than physical aggression. To promote maternal perinatal mental health, clinicians should screen for covert forms of psychological aggression during pregnancy (e.g., raised voices, insults), trauma-related distress, and symptom elevations in women and their partners.

## 1. Introduction

Intimate partner violence (IPV) affects many couples worldwide and is primarily studied in the forms of psychological aggression (e.g., insulting, shouting at, or swearing at a partner) and physical aggression (e.g., pushing, slapping, or punching a partner) [1,2]. In the United States (US), over one in three women experience some form of IPV during their lifetimes, with upwards of 6 million women experiencing IPV each year [3]. Rates of psychological aggression, which may be especially detrimental to mental health, are particularly high, with an estimated 75–80% of couples engaging in psychologically aggressive acts during arguments [4,5,6]. IPV rates during pregnancy, a period of heightened risk [7], are nearly identical to lifetime rates [8]. Further, research suggests psychological aggression is most the prevalent form of IPV during the perinatal period [9].

Pregnancy represents a period of substantial risk for depression and comorbid disorders, with rates of perinatal depression ranging from 13–19% [10,11]. Importantly, the mental health of parents during pregnancy has implications for the health of the family after the child is born. Maternal prenatal depression and anxiety have significant consequences for fetal development and birth outcomes [12,13]. Prenatal depression is also the strongest predictor of postpartum depression [14], which negatively impacts parental mental and physical health, parent–child interactions, and infant development [15].

Although research has largely focused on maternal symptomology, fathers are also at increased risk for depression during pregnancy and the postpartum period [16,17]. As such, identifying critical risk factors for maternal and paternal depression and anxiety during pregnancy, which sets the stage for problems during the postpartum period, is a critical research endeavor. The present study sought to build on existing research demonstrating the detrimental impact of IPV on mental health during pregnancy [8,12,18] by (1) examining both maternal and paternal internalizing symptoms and (2) investigating whether psychological and physical aggression are uniquely associated with not only the broad negative affectivity underlying depression and anxiety but also specific manifestations of internalizing pathology (e.g., panic, social anxiety).

### 1.1. Intimate Partner Violence as a Risk Factor for Depression and Anxiety

Both psychological and physical aggression in intimate relationships impact mental health [19], including risk for depression and anxiety, as well as posttraumatic stress disorder (PTSD) [20,21,22]. Extant research examining the effect of intimate partner aggression on women demonstrates consistent positive associations between IPV and depression and anxiety symptomology [21]. The risk of maternal depression and anxiety is already elevated during pregnancy and the postpartum period [23], and pregnant women who experience IPV are 2.5 times more likely to have depressive symptoms than those who do not experience IPV [12]. In fact, up to 46% of pregnant women who experience IPV demonstrate clinically significant levels of depression [24]. Research also highlights the importance of considering the type of IPV unfolding in couple relationships. In community samples, psychological aggression is uniquely associated with mental health difficulties (e.g., depressive and anxiety symptoms) and relationship dissatisfaction when controlling for physical aggression [4,5,6,25]. Research also highlights the deleterious effects of psychological aggression—the most prevalent form of IPV during the perinatal period—for pregnant women [26].

Although most research has examined IPV perpetrated against women, understanding how IPV undermines the mental health of men is also an important empirical endeavor given the implications of paternal depression and anxiety for the health of the family (e.g., maternal mental health, parent–infant bonding, parenting, and child outcomes) [27]. Research on IPV experienced by men has produced inconsistent findings; some research suggests the link between IPV and depression is similar for men and women, while other research suggests this association is non-significant for men [4,20]. Further, to our knowledge, there is no research on the impact of IPV during pregnancy on paternal psychopathology despite the importance of fathers’ mental health during this unique time in the family lifecycle [28].

Finally, in community samples of couples, perpetration of psychological and physical intimate partner aggression has been found to be largely bidirectional [4,20,29]. Despite these findings, most studies of IPV include reports from only one partner of a dyad [30]. Thus, it is imperative that IPV research is conducted within a dyadic framework to best understand the consequences of being in a relational context characterized by a cycle of aggressive acts during arguments. Indeed, Mojahed and colleagues highlight the need for research examining the occurrence of bidirectional IPV unfolding within the couple relationship—not just victimization at the individual level—during the perinatal period along with the detrimental consequences of IPV within the family system [9]. Moreover, as research increasingly demonstrates that the perinatal period is associated with complex mental health changes beyond depressive symptoms alone [31,32], it is important to look at the consequences of IPV within a broader and more integrative framework of psychopathology.

### 1.2. Embracing a Hierarchical Framework of Internalizing Disorders

Comorbidity across mood and anxiety disorders has been particularly well-documented in general and perinatal populations [33,34]. Studies examining multivariate models of the interrelations of psychological disorders suggest that a hierarchical model provides the best organizing framework for understanding psychopathology. Based on this evidence, the Hierarchical Taxonomy Of Psychopathology (HiTOP) consortium has articulated a comprehensive hierarchical, dimensional model of symptoms and syndromes [35]. Within the HiTOP model, symptoms of similar nature are grouped into syndromes (disorders) based on their natural covariation, and correlated syndromes are explained by overarching subfactors. These subfactors are likewise combined into spectra, which represent the higher-order dimensions of psychopathology. Moreover, the HiTOP model highlights the dimensional assessment of psychopathology. Dimensional models are particularly useful for measuring constructs without clear boundaries [36] and provide information beyond the presence versus absence of a disorder [37]. Examining the symptoms of a disorder may be more reliable than depending on diagnostic categories and can yield incremental information [38].

Within the HiTOP model, the comorbidity between depressive and anxiety disorders can be explained by the shared higher-order factor of *negative affectivity* [39,40]. This broad internalizing spectrum can be further broken down into two highly correlated subfactors, which have been labeled “distress” (marked by major depression, dysthymia, PTSD, and generalized anxiety) and “fear” (marked by panic disorder, agoraphobia, social anxiety, obsessive compulsive disorder, and specific phobia) [39,41,42,43]. This model explains both the comorbidity (between mood and anxiety symptoms) and heterogeneity (across the various types of anxiety symptoms) that define the internalizing spectrum by isolating the common and unique features of each disorder.

Although IPV is a robust predictor of depression, past research has not consistently accounted for the comorbidity among internalizing disorders within a unified model [44]. Thus, the HiTOP model may prove especially useful for understanding the association between IPV and psychopathology, as it permits an examination of the level at which IPV constitutes a risk factor [45]. One possibility is that IPV is a non-specific risk factor that increases the likelihood that an individual could develop a range of psychopathologies. Alternatively, IPV may be a risk factor for particular forms of pathology, such as depression or PTSD [21,22]. By assessing IPV within a hierarchical model, researchers can observe whether IPV confers risk for internalizing disorders in general, for certain disorders, or even for specific symptoms.

### 1.3. The Present Study

The primary objective of the present study was to investigate psychological and physical aggression unfolding in couple relationships and their associations with the mental health of mothers and fathers during pregnancy. The first aim was to examine the unique effects of psychological and physical intimate partner aggression on the higher-order shared dimension of negative affectivity common to depressive and anxiety disorders. We predicted that higher levels of psychological aggression in the couple relationship would be uniquely associated with negative affectivity when controlling for physical aggression and other salient demographic risk factors for IPV and psychopathology (i.e., age and ethnicity of each partner, relationship duration, annual joint income, week of pregnancy) [46,47,48,49]. We anticipated that this unique link would emerge for both women and men and would be of a similar magnitude, demonstrating the equally detrimental impact of psychological aggression for both pregnant mothers and their partners.

The second aim, which was exploratory in nature, examined the unique effects of psychological and physical intimate partner aggression on specific manifestations of internalizing problems (i.e., the specific symptoms associated with mood and anxiety disorders). Based on past work on PTSD, we hypothesized that higher levels of psychological and physical aggression in the couple relationship would be associated with higher levels of trauma-related distress (i.e., avoidance and intrusions) in both partners. We did not make specific predictions for the remaining internalizing symptoms.

The current study builds on existing literature by using a hierarchical model of internalizing disorders as an organizing framework, allowing us to look at both the higher-order shared features of these disorders as well as the lower-order specific manifestations of each syndrome, and assessing disorders dimensionally by measuring the severity of symptoms of each disorder rather than categorical diagnoses. Further, this study aims to identify potential risk factors for poor mental health in both mothers and fathers during pregnancy. Finally, building on past research suggesting that psychological aggression might be more detrimental to mental health than physical aggression [4], we examined the incremental effects of psychological and physical aggression controlling for other salient risk factors for psychopathology.

## 2. Materials and Methods

Procedures for the present study were approved by the University of Nebraska-Lincoln Institutional Review Board. Eligibility criteria for a larger study of couples navigating pregnancy included: (a) 19 years of age or older, (b) English speaking, (c) pregnant at the time of the initial appointment, (d) both biological parents of the child, (e) singleton pregnancy, and (f) in a committed intimate relationship and cohabiting. A total of 162 couples enrolled in the study, but 3 couples were excluded from the final sample due to either ineligibility or invalid data, leading to a final sample of 159 couples (159 women and 159 men). Couples dated for an average of 81.90 months (*SD* = 49.59). Most couples were married (84.9%). Women were primarily in the second (38.4%) or third (58.5%) trimester of pregnancy. Most participants were White (89.3% of females; 87.4% of males), while 9.4% of women and 6.4% of men identified as Hispanic or Latino/a. Women had an average age of 28.67 years (*SD* = 4.27), and men had an average age of 30.56 years (*SD* = 4.52). Annual joint income ranged from less than USD 9999 to more than USD 90,000, with a median joint income of USD 60,000 to USD 69,999. Modal education was a bachelor’s degree (46.5% of women; 34.6% of men), and most participants were employed at least 16 h per week (74.2% of women; 91.8% of men). Both partners attended a laboratory appointment, during which they completed self-report questionnaires on laptops in private rooms, separate from one another, using Qualtrics software (Provo, UT, USA). Participants entered a study identification number but no other identifying information. Participants were compensated with USD 50 (for a total of USD 100 per couple).

### 2.1. Measures

Psychological and Physical Intimate Partner Aggression in the Couple Relationship. The Short Form of the *Revised Conflict Tactics Scales* (CTS-Short Form) is a self-report questionnaire that assesses tactics used during conflicts in intimate relationships, including different manifestations of partner violence [50]. Previous studies demonstrate that the short form is similar in validity to the full version [50]. The CTS-Short Form includes two psychologically aggressive behaviors (“My partner insulted or swore or shouted or yelled at me” and “My partner destroyed something belonging to me or threatened to hit me”) and two physically aggressive behaviors (“My partner pushed, shoved, or slapped me” and “My partner punched or kicked or beat-me-up”). Each behavior is asked in terms of perpetration (e.g., “I punched or kicked or beat up my partner”) and victimization (e.g., “My partner punched or kicked or beat me up”), for a total of 8 items.

Participants were asked to rate each item in terms of the frequency it occurred in the relationship *over the past year*. Consistent with procedures used by Kan and Feinberg [51], we scored the frequency of a given behavior by examining reports from both partners and selecting the maximum estimate of violence directed toward one partner (e.g., highest reported frequency) to address potential underreporting. For example, if a mother reported her partner pushed, shoved, or slapped her 3–5 times, but her partner reported he had only enacted that behavior toward her once, we coded that act as occurring 3–5 times toward mother. Frequency ratings made by participants were recoded based on published scoring guidelines: once = 1, twice = 2, 3–5 times = 4, 6–10 times = 8, 11–20 times = 15, more than 20 times = 25.

*Frequency of psychological aggression in the relationship over the past year.* In the present study, the frequency of psychological aggression directed toward each partner ranged from 0 to 33 (*M* = 6.07, *SD* = 8.15 for aggression toward mothers; *M* = 7.42, *SD* = 8.92 for aggression toward fathers), with 75.5% of couples reporting any instance of psychological aggression. As expected in this sample of pregnant couples, maternal and paternal frequency scores of psychological aggression victimization were highly correlated (*r* = 0.83, *p* < 0.001); thus, a dyad-level score was computed representing the average frequency of psychological aggression occurring in the relationship during the past year. This scoring method, which characterizes aggression at the couple level, also reflects the bidirectional nature of aggression that is common in community samples of couples [4,20,29].

*Any incident of physical aggression in the relationship over the past year.* The frequency of physical aggression was relatively rare in this sample; on average, less than one act of physical aggression occurred over the past year (*M* = 0.57, *SD* = 2.68 for aggression toward mothers; *M* = 0.98, *SD* = 3.39 for aggression toward fathers). Due to low rates of physical aggression, a binary score was computed representing whether any physical aggression had been directed toward each partner within the past year—approximately 10% of mothers and 17% of fathers were victims of physical aggression. Maternal and paternal binary scores of physical aggression victimization were highly correlated (Spearman *r* = 0.74, *p* < 0.001), once again capturing IPV at the couple level (not just at the level of individual victimization) and reflecting the largely bidirectional nature of physical aggression that was expected in this community sample of couples. Consequently, a dyad-level score of physical aggression was computed such that 1 = any physical aggression occurring in the past year (by either or both partners; 17% of couples) and 0 = no physical aggression in the relationship. Notably, all couples reporting physical aggression also reported at least one instance of psychological aggression.

Hierarchical Model of Internalizing Disorders. The Expanded Form of the *Inventory of Depression and Anxiety Symptoms* (IDAS-II) was used to assess internalizing psychopathology at the symptom level of the hierarchy [52]. The IDAS-II contains multiple symptom scales. Respondents rate their feelings and experiences during the past two weeks on a scale from one (not at all) to five (extremely). For Aim 1, we focused on the 10-item Dysphoria scale, which assesses the nonspecific emotional and cognitive symptoms of depression and anxiety, capturing the shared negative affectivity factor of the hierarchical model of internalizing disorders [40,53]. The internal consistency of the scale was adequate (Cronbach’s alpha = 0.83). For Aim 2, we examined a subset of 9 of the 17 factor-analytically derived specific symptom scales of the IDAS-II: Well-Being (8 items), Lassitude (6 items), Insomnia (6 items), Ill Temper (5 items), Panic (8 items), Social Anxiety (6 items), Claustrophobia (5 items), Traumatic Intrusions (4 items), and Traumatic Avoidance (4 items). These scales are internally consistent and stable, demonstrate good convergent and discriminant validity, provide incremental predictive power beyond existing measures in identifying clinical diagnoses, and differentiate psychiatric patients from nonclinical participants [52,54,55,56]. Thus, the IDAS-II uniquely allows for the assessment of the hierarchical structure of internalizing symptoms, in line with the HiTOP model [35]. In the present study, the specific scales demonstrated adequate internal consistency (alphas ranged from 0.75 to 0.88).

In selecting which scales to include in the present analyses, we prioritized symptoms that (a) would likely not be attributed to the physical symptoms characteristic of pregnancy and (b) are clear markers of the higher-order internalizing domain. Nylen and colleagues examined the validity of the original IDAS symptom scales as indicators of depression during pregnancy and found that, whereas Insomnia and Lassitude identified depression above and beyond their shared variance as common pregnancy experience, Appetite Gain and Loss—which could be due to physical changes during pregnancy—did not [57]. Therefore, we excluded the two appetite scales from the analysis. We also chose to exclude OCD symptoms (Checking, Ordering, Cleaning) and mania symptoms (Mania, Euphoria), given increasing evidence that these symptoms are indicators of the thought disorder dimension and are weaker markers of internalizing disorders [35]. Finally, we excluded Suicidality due to extremely low rates in this sample (e.g., two of the items were uniformly endorsed as “not at all”, and only 1.3% of participants endorsed any thought of suicide). By focusing on this subset of theoretically meaningful scales, we were also able to prioritize model parsimony in dyadic analyses conducted with this relatively modest sample size.

Control Variables. We measured several characteristics of each partner and the relationship to include as controls. Younger age, lower income, ethnic minority status, and shorter relationship duration are established risk factors for IPV, including during pregnancy [46,49]. In addition, research points to systematic mood fluctuations throughout the course of pregnancy [58], and income and week of pregnancy are also related to prenatal depressive symptoms [47,48]. Therefore, the age of each partner, ethnicity of each partner, annual joint income, relationship duration, and week of pregnancy at the time of assessment were used as controls in the analyses.

### 2.2. Data Analytic Approach

The aims were pursued using path analysis in Mplus [59]. Missing data were minimal and were addressed with a maximum likelihood estimation (covariance coverage ranged from 0.99 to 1.00 across the tested models) [60]. Further, we implemented a robust maximum likelihood estimator (MLR), which produces standard errors of parameter estimates that are robust to non-normality. Maternal and paternal IDAS scores were modeled as separate variables in the same model consistent with contemporary approaches to analyzing data from distinguishable dyads [61]; residuals were correlated across partners to account for interdependence across members of a dyad (e.g., maternal and paternal reports of dysphoria).

## 3. Results

Descriptive statistics for the IDAS-II scales are reported in Table 1. Paired-sample t-tests were conducted to test whether there were significant mean differences in maternal and paternal scores. On average, relative to men, pregnant women scored higher on lassitude, insomnia, and panic. In contrast, men had higher scores on traumatic avoidance. Interpartner correlations are also reported in Table 1 to evaluate whether symptoms reported by one partner were significantly associated with symptoms reported by the other partner; correlations were significant for dysphoria, along with ill temper, claustrophobia, and traumatic avoidance.

Correlations between each form of aggression and the IDAS-II scales are reported in Table 2 for mothers and fathers. Both psychological and physical aggression were correlated with dysphoria for both mothers and fathers. Regarding specific symptom dimensions for mothers, psychological and physical aggression were associated with higher levels of trauma-related distress (both intrusions and avoidance) and ill temper; physical aggression was also associated with panic and claustrophobia. For fathers, psychological aggression was significantly associated with every scale in the expected directions, whereas physical aggression was associated with greater insomnia, ill temper, panic, and traumatic intrusions.

**Figure 1:**
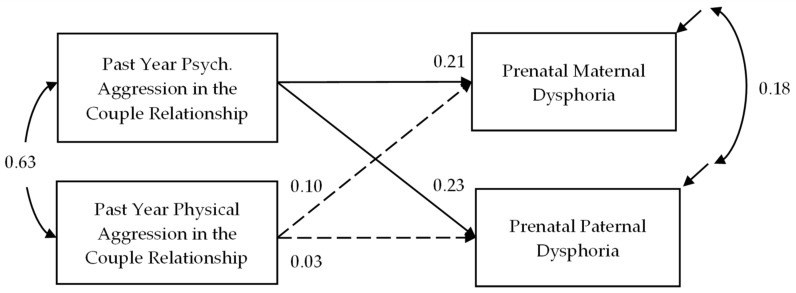
Psychological (Psych.) Aggression is Uniquely Associated with Negative Affectivity (Dysphoria Scale of the IDAS-II). Standardized estimates. Significant effects (*p* < 0.05) are depicted with a solid line, and non-significant effects are depicted with a dashed line. This model was just identified. The week of pregnancy, age of each partner, ethnicity of each partner, annual joint income, and relationship duration were included as covariates in the model but are omitted from the figure for ease of presentation.

**Aim 1.** Results of Aim 1 are reported in Figure 1. Only psychological aggression was uniquely associated with the broad dimension of negative affectivity common to internalizing disorders when controlling for physical aggression and a range of demographic characteristics. Psychological aggression was associated with both maternal and paternal dysphoria, and a Wald chi-square test suggests the effect was not significantly larger for fathers (β = 0.23) relative to mothers (β = 0.21), χ^2^ (1) = 0.24, *p* = 0.63.

**Aim 2.** This exploratory aim revealed specific symptom dimensions most strongly relating to intimate partner aggression (see Table 3). For mothers, psychological aggression was uniquely associated with ill temper and traumatic intrusions. For fathers, psychological aggression was uniquely associated with lassitude, ill temper, and traumatic avoidance.

## 4. Discussion

The goal of the present study was to examine the association between past psychological and physical aggression unfolding in the couple relationship and the mental health of mothers and fathers during pregnancy within a hierarchical model of internalizing disorders. Consistent with past research, more frequent psychological aggression in one’s intimate relationship, and any occurrence of physical aggression, were associated with higher levels of negative affectivity during pregnancy for both women and men. When testing a unified model with both forms of aggression modeled simultaneously—and controlling for demographic risk factors for IPV and depression—only psychological aggression emerged as uniquely related to maternal negative affect. These findings are consistent with previous research linking IPV to maternal depression during pregnancy [12,24] and build on this work by identifying risks for a general dimension of depressive and anxiety symptoms thought to represent a broad liability for internalizing disorders, thus demonstrating the far-reaching implications of psychological aggression for maternal mental health. The results also converge with research, suggesting that psychological aggression might be especially detrimental to the mental health of women, more so than physical aggression [4,5,6].

Although IPV research often focuses on women [21], our results demonstrate that IPV is also associated with greater negative affectivity experienced by men during pregnancy. Consistent with the results for women, psychological aggression also emerged as uniquely detrimental for men, and the effect was of similar magnitude. Further, paternal reports of negative affectivity were significantly correlated with maternal negative affectivity—in addition to several specific symptom dimensions (i.e., traumatic avoidance, ill-temper, claustrophobia)—suggesting that promoting the mental health of fathers during pregnancy might also serve to promote maternal mental health. This highlights the critical need for couple-level interventions during the perinatal period rather than focusing solely on expecting mothers.

We also pursued an exploratory aim that further capitalized on the hierarchical measurement of depressive and anxiety disorders during pregnancy, and results suggest that intimate partner aggression might be associated with unique manifestations of symptoms. Results suggest that women in relationships characterized by psychological aggression are prone to experience not only general negative mood (dysphoria) but also specific symptom manifestations (i.e., ill temper and trauma-related distress) when controlling for physical aggression and demographic variables. For men, psychological aggression was uniquely associated with lassitude, ill temper, and trauma-related distress in the form of avoidance. This pattern of results converges with research suggesting that IPV can represent a form of traumatic exposure that increases the risk for PTSD [62] and demonstrates the unique role that psychological aggression might play in the development of PTSD. In addition, both mothers and fathers in psychologically aggressive relationships were prone to experiencing greater ill temper (anger/hostility), and men reported greater lassitude (fatigue) and symptoms associated with PTSD [55,63].

Finally, it was notable that, when examining correlations (Table 2), men demonstrated a more heterogeneous symptom presentation associated with IPV; psychological aggression was associated with every dimension of internalizing symptoms—including general negative affectivity and specific symptoms of lassitude, insomnia, low well-being, ill temper, panic, social anxiety, traumatic intrusions, and traumatic avoidance. Thus, in addition to experiencing symptoms consistent with PTSD, which largely characterized symptom presentation for women, men experienced other notable mood disturbances and anxiety symptoms. Consequently, screening and detection of internalizing problems resulting from IPV might be more challenging for men, given symptoms can manifest in disparate ways.

### 4.1. Limitations

Several limitations to the present study should be considered when interpreting the results. First, a primary limitation of the study is the generalizability of the results. Our community sample of pregnant couples had low levels of internalizing psychopathology relative to clinical samples; mean values were generally comparable to the community, pregnant, and postpartum samples [52,56,57]. It is possible that results would vary in a sample of participants experiencing clinically significant levels of depression; however, it is important to note that subthreshold anxiety and depression symptoms are not uncommon, are associated with functional impairment, and are predictors of later development of major depressive and anxiety disorders [64,65,66]. Future work should incorporate a clinical sample of pregnant couples, which would additionally allow the assessment of the low base-rate symptoms we excluded in the present analysis (e.g., suicidality). Consistent with demographics in the region where the research was conducted, the sample was comprised of heterosexual couples who were predominantly White, well-educated, and employed. This further limited generalizability. Low socioeconomic status (SES) is associated with increased risk of IPV, and the relative influence of SES on IPV varies across racial and ethnic groups [67,68]. Research is needed to understand how associations between IPV and internalizing symptoms vary as a function of demographic characteristics in more diverse samples.

Second, social desirability could lead to underreporting IPV; however, recent research suggests social desirability effects are not as problematic as once expected [69]. Nonetheless, we examined reports from both partners of a dyad and retained the report of highest frequency for a particular behavior to reduce potential reporting bias. Further, reports of aggression and internalizing symptoms were assessed concurrently, and conclusions about the directionality of effects should be interpreted with caution, given depression can predict aggressive behavior [20]. This concern is mitigated by the assessment of aggression “during the past year” and internalizing symptoms over the past “2 weeks,” providing some degree of temporal precedence of aggression relative to depressive symptoms; however, given the cross-sectional nature of the data, associations between IPV and internalizing symptoms might be smaller when employing longitudinal designs with temporal lags between study variables. Finally, rates of physical aggression were relatively low in this community sample of couples, and future research might oversample for couples engaging in more persistent patterns of physical IPV to better understand the link between IPV and mental health during pregnancy.

Finally, Aim 2 was exploratory in nature and was intended to serve the purpose of generating hypotheses for future research. Nonetheless, the possibility of Type I errors due to the number of effects estimated across the various symptom dimensions should be considered, and replication is required. It was notable that the largest correlations emerged for ill temper in relation to psychological aggression, *r* = 0.32 for women and *r* = 0.44 for men. As such, ill temper appears to be a particularly salient dimension of internalizing symptoms to investigate in future research on intimate partner aggression during pregnancy for both men and women, especially given ill temper might undermine effective conflict management and resolution strategies, perpetuating a cycle of IPV.

### 4.2. Research Implications

The present study has implications for conceptual frameworks of IPV and mental health and research aimed at understanding the consequences of aggression in intimate relationships for individual partners, particularly among community samples with low overall levels of psychopathology. Taken together, the results highlight the utility of assessing symptoms using a hierarchical conceptualization of internalizing disorders such as the HiTOP model to identify typical symptom profiles emerging in response to IPV for men versus women during pregnancy. Further, the results suggest that intimate partner aggression—and psychological aggression in particular—may be a common risk factor for the Internalizing spectrum. This demonstrates that the IPV-mental health association is not specific to one disorder, such as major depression, and research efforts aiming to understand the role of IPV in psychopathology could be strengthened by utilizing a hierarchical framework, such as those proposed by the HiTOP model. Indeed, close attention should be paid to the impact of IPV, especially psychological aggression, on trauma-related distress during pregnancy in the form of re-experiencing symptoms (e.g., nightmares, flashbacks) and avoidance of internal and external stimuli associated with trauma. Trauma-related distress has the potential to be especially damaging to pregnant women, given that exposure to trauma may moderate the effect of childbirth on maternal mental health or exacerbate pre-existing trauma symptoms [70].

### 4.3. Clinical Implications

Collectively, our results have implications for prenatal screening and intervention efforts aimed at interrupting the maladaptive cycle of IPV and promoting the mental health of mothers and their partners during pregnancy. First, the results suggest that it is important to routinely screen for IPV during pregnancy and provide appropriate referrals; however, exclusively screening for physical aggression and the victimization of women may be insufficient. Instead, attention should be paid to arguments that are characterized by raised voices, insults, and threats displayed by both partners, not just physical acts, such as pushing or shoving. From a prevention standpoint, teaching conflict management skills during prenatal classes could be beneficial. Interventions that focus on enhancing communication and conflict management skills as a couple during pregnancy, such as the *Family Foundations Program* [71], have the potential to reduce IPV and parental psychopathology, strengthen the couple relationship and promote resiliency, and ultimately improve child outcomes. Further, given that women and their partners engage with the healthcare system more regularly across the prenatal period, models of integrated perinatal care may be particularly beneficial for addressing IPV and associated psychopathology by increasing access to care and providing more timely services [72,73]. In addition, efforts to address paternal perinatal mental health are critical for maternal well-being, as untreated psychopathology may interfere with fathers’ ability to support their partners across pregnancy and the transition to parenthood [28]. Thus, current approaches to perinatal mental health care must be expanded to include more routine screening for paternal psychopathology, which would facilitate appropriate referrals, and involve both mothers and fathers in treatment.

Second, psychological aggression and physical aggression were associated with internalizing symptoms for both men and women, which is especially notable given the relative dearth of research on the mental health consequences of IPV for men, particularly during pregnancy. Existing IPV interventions for fathers tend to focus on programs designed to deal with anger management, victim protection, etc., or are focused on the father–child interaction and parenting techniques rather than treating underlying psychopathology [74]. Given that offender intervention programs are associated with high dropout rates [75], it is imperative that fathers’ mental health issues be addressed, such that their contribution to the IPV cycle can be disrupted, rather than relying on treatments focusing solely on the aggressive behaviors. For instance, building on evidence that experiential avoidance may increase the potential for aggressive behavior, recent work suggests that group-based Acceptance and Commitment Therapy (ACT) may be particularly helpful in reducing partner aggression [76].

## 5. Conclusions

The present study demonstrated that more frequent psychological aggression during arguments between intimate partners and any occurrence of physical aggression were associated with higher levels of negative affectivity during pregnancy not only for women but also their partners. However, only psychological aggression demonstrated incremental prediction, suggesting that arguments characterized by raised voices and insults might be particularly harmful to perinatal mental health despite the potential to downplay the consequences of these behaviors relative to physically aggressive acts. Further, our application of a hierarchical model of internalizing disorders extends past work largely focused on perinatal depression by identifying the unique symptom manifestations associated with IPV. The results highlight that pregnant women might be particularly susceptible to experiencing trauma-related distress, which has the potential to be further exacerbated by the childbirth experience, highlighting the need for practitioners to routinely screen for avoidance and intrusions related to past trauma. Finally, symptom elevations associated with IPV were observed in not only women but also their partners. Thus, the results underscore the importance of couple-level interventions that not only promote healthy relational dynamics during pregnancy, including constructive conflict management skills (e.g., *Family Foundations Program*) but also consider the mental health of both partners in dual parenting households.

## Figures and Tables

**Table 1:** Descriptive Statistics for IDAS-II Scales for Mothers and Fathers during Pregnancy.

IDAS-II Scale	Maternal		Paternal		Paired *t* (df)	Paired *r*
	M	SD	M	SD		
Dysphoria	17.03	5.07	16.37	5.83	1.24 (158)	0.25 **
Lassitude	**12.93**	**4.42**	10.89	3.91	4.73 (158) **	0.15
Insomnia	**14.08**	**4.95**	11.12	4.70	5.59 (158) **	0.04
Well-Being	27.36	4.75	26.36	5.37	1.83 (158)	0.07
Ill Temper	6.67	2.27	6.92	2.98	−0.93 (158)	0.17 *
Panic	**10.60**	**3.06**	9.23	2.72	4.37 (158) **	0.06
Social Anxiety	8.16	3.24	8.59	3.59	−1.17 (158)	0.06
Claustrophobia	5.46	1.69	5.84	2.59	−1.75 (158)	0.25 **
Traumatic Intrusions	5.21	2.15	5.02	1.98	0.88 (158)	0.15
Traumatic Avoidance	5.19	2.16	**5.83**	**3.16**	−2.33 (158) *	0.19 *

Note. In the case of a significant mean difference between maternal and paternal scores, the higher mean is bolded. * *p* < 0.05. ** *p* < 0.01.

**Table 2:** Correlations between Aggression in the Couple Relationship and IDAS-II Scales.

	Maternal		Paternal	
	Psychological	Physical	Psychological	Physical
Dysphoria	**0.27** **	**0.24** **	**0.28** **	**0.19** *
Lassitude	0.06	0.14	**0.26** **	0.15
Insomnia	−0.01	0.01	**0.27** **	**0.24** **
Well-Being	−0.09	−0.07	**−0.17** *	−0.14
Ill Temper	**0.32** **	**0.30** **	**0.44** **	**0.23** **
Panic	0.09	**0.23** **	**0.21** **	**0.23** **
Social Anxiety	0.14	0.12	**0.24** **	0.11
Claustrophobia	0.15	**0.19** *	**0.22** **	0.11
Traumatic Intrusions	**0.24** **	**0.25** **	**0.27** **	**0.24** **
Traumatic Avoidance	**0.23** **	**0.26** **	**0.32** **	0.20 *

Note. Pearson correlations are reported for the frequency of psychological aggression and IDAS-II scales. Point biserial correlations are reported for physical aggression (1 = any in past year, 0 = none) and IDAS-II scales. * *p* < 0.05. ** *p* < 0.01. Bold indicates a statistically significant correlation (*p* < 0.05).

**Table 3:** Psychological Aggression is Uniquely Associated with Specific Depressive and Anxiety Symptoms.

	Maternal				Paternal			
	b	SE	*p*-Value	β	b	SE	*p*-Value	β
*Lassitude*								
Psychological	−0.03	0.06	0.583	−0.06	**0.11**	**0.05**	**0.019**	**0.24**
Physical	1.69	1.45	0.245	0.14	−0.24	1.12	0.834	−0.02
*Insomnia*								
Psychological	0.01	0.07	0.886	0.02	0.11	0.07	0.088	0.20
Physical	0.57	1.56	0.716	0.04	0.85	1.37	0.537	0.07
*Well-Being*								
Psychological	−0.03	0.06	0.609	−0.06	−0.09	0.07	0.201	−0.13
Physical	−0.19	1.56	0.904	−0.02	−0.91	1.46	0.535	−0.06
*Ill Temper*								
Psychological	**0.06**	**0.03**	**0.040**	**0.21**	**0.17**	**0.05**	**0.002**	**0.46**
Physical	0.92	0.66	0.162	0.15	−0.95	1.05	0.366	−0.12
*Panic*								
Psychological	−0.02	0.03	0.529	−0.06	0.02	0.03	0.340	0.07
Physical	1.48	0.97	0.128	0.18	0.94	0.97	0.332	0.13
*Social Anxiety*								
Psychological	0.06	0.03	0.093	0.14	0.11	0.06	0.063	0.24
Physical	−0.30	0.75	0.690	−0.04	−0.27	1.11	0.810	−0.03
*Claustrophobia*								
Psychological	0.02	0.03	0.514	0.09	0.07	0.05	0.134	0.22
Physical	0.20	0.54	0.710	0.05	−0.54	0.86	0.529	−0.08
*Traumatic Intrusions*								
Psychological	**0.05**	**0.03**	**0.031**	**0.20**	0.04	0.02	0.125	0.15
Physical	0.03	0.51	0.949	0.01	0.36	0.58	0.535	0.07
*Traumatic Avoidance*								
Psychological	0.04	0.03	0.145	0.15	**0.12**	**0.05**	**0.015**	**0.31**
Physical	0.35	0.56	0.529	0.06	−0.70	0.99	0.478	−0.08

Note. This model was just identified. Significant effects (*p* < 0.05) are bolded. Week of pregnancy, age of each partner, ethnicity of each partner, annual joint income, and relationship duration were included as covariates in the model but are omitted for ease of presentation.

## Data Availability

Data are available upon request to the corresponding author, pending an approval process through IRB.
